# Beyond microarrays: Finding key transcription factors controlling signal transduction pathways

**DOI:** 10.1186/1471-2105-7-S2-S13

**Published:** 2006-09-26

**Authors:** Alexdander Kel, Nico Voss, Ruy Jauregui, Olga Kel-Margoulis, Edgar Wingender

**Affiliations:** 1 BIOBASE GmbH, Halchtersche Str. 33, D-38304 Wolfenbüttel, Germany; 2 Dept. Bioinformatics, UKG/Univ. Göttingen, Goldschmidtstr. 1, D-37077 Göttingen, Germany

## Abstract

**Background:**

Massive gene expression changes in different cellular states measured by microarrays, in fact, reflect just an "echo" of real molecular processes in the cells. Transcription factors constitute a class of the regulatory molecules that typically require posttranscriptional modifications or ligand binding in order to exert their function. Therefore, such important functional changes of transcription factors are not directly visible in the microarray experiments.

**Results:**

We developed a novel approach to find key transcription factors that may explain concerted expression changes of specific components of the signal transduction network. The approach aims at revealing evidence of positive feedback loops in the signal transduction circuits through activation of pathway-specific transcription factors. We demonstrate that promoters of genes encoding components of many known signal transduction pathways are enriched by binding sites of those transcription factors that are endpoints of the considered pathways. Application of the approach to the microarray gene expression data on TNF-alpha stimulated primary human endothelial cells helped to reveal novel key transcription factors potentially involved in the regulation of the signal transduction pathways of the cells.

**Conclusion:**

We developed a novel computational approach for revealing key transcription factors by knowledge-based analysis of gene expression data with the help of databases on gene regulatory networks (TRANSFAC^® ^and TRANSPATH^®^). The corresponding software and databases are available at .

## Background

New high-throughput methods, such as microarrays, allow generation of massive amounts of molecular biological data. These, mainly phenomenological, data are often difficult to relate with the activation/inhibition of particular signal transduction pathways and/or transcriptional regulators. Gene expression changes in different cellular states measured by microarrays, in fact, reflect just an "echo" of real molecular processes in the cells. A way to facilitate data interpretation is to construct gene regulatory networks that include signal transduction mediators, transcriptional regulators and target genes. This is a complex task, not only because of the huge number of molecules involved, but also because of variations across tissues, developmental stages and physiological conditions. However, these networks hold the key to the understanding of the regulatory processes within a cell and, thus, to the majority of life processes in general.

Changes of expression of genes encoding transcription factors (TFs), a class of key regulatory molecules, are often hard to reveal to be significantly up- or downregulated in microarray experiments since their expression changes are small and their activity is mainly regulated on the posttranscriptional level. Analysis of promoters of co-expressed genes can provide one source of evidences on involvement of certain TFs in the regulation of the genes. Several computational approaches have been developed in the past few years in order to reveal potential binding sites in the promoter regions of co-expressed genes. They applied various techniques ranging between simple pattern search and complex models such as HMMs (Hidden Markov Models). The most widely used method is based on positional weight matrices (PWMs) that are constructed from collections of known binding sites for given TF or TF family. One of the largest collections of TF binding sites (TFBS) and corresponding PWMs is the TRANSFAC^® ^database [1]. The PWM approach was applied intensively in the last years for the analysis of regulatory regions of many different functional classes of genes, for instance, globin genes [2], muscle- and liver-specific genes [3,4], and cell cycle-dependent genes [5]. In recent approaches, in order to improve the site prediction quality, different authors have searched for combinations of TFBS – cis-regulatory modules [6-10] and have applied comparative genomics approaches [11-13]. Despite these efforts, understanding the full complexity of the gene regulatory regions remains a great challenge and it is still rather problematic to identify transcription factors involved in the regulation of genes under any particular cellular condition based on the promoter analysis alone.

Another source of evidences on the key role of transcription factors in regulating cellular regulatory processes comes from analysis of signal transduction pathways. Multiple signal transduction pathways of a cell transduce extracellular signals from receptors at the cellular membrane to the transcription factors in the nucleus where they regulate the transcription of genes. There are several databases that collect information about signal transduction pathways in different cells. Among them, the TRANSPATH^® ^database [14] stores a large body of information on signaling pathways allowing computational search through the graph of signaling reactions. One aim of such searches is to find the key transcription factors that mediate the concerted changes in expression of specific components of the signal transduction network.

In this paper we report an attempt to integrate the two complementary approaches for identification of key TFs: 1) analysis of promoters of co-expressed genes and 2) analysis of networks of the differentially expressed components of the signal transduction pathways. We have developed two computational tools: *F-Match*™ for revealing over- and underrepresented sites of promoters and *ArrayAnalyzer*™ for identification of key nodes in signal transduction networks. The developed integrated approach aims to reveal multiple evidences of positive feedback loops in signal transduction circuits through activation of pathway-specific transcription factors.

We demonstrated that promoters of genes encoding components of many known signal transduction pathways are enriched by binding sites of those transcription factors that are end-points of the considered pathways. Application of the approach to the microarray gene expression data on TNF-alpha stimulated primary human endothelial cells [15] helped to reveal novel key transcription factors that are potentially involved in the regulation of the signal transduction pathways of these cells.

## Results

### TFBS in promoters of genes belonging to the same pathway

**Figure 1:**
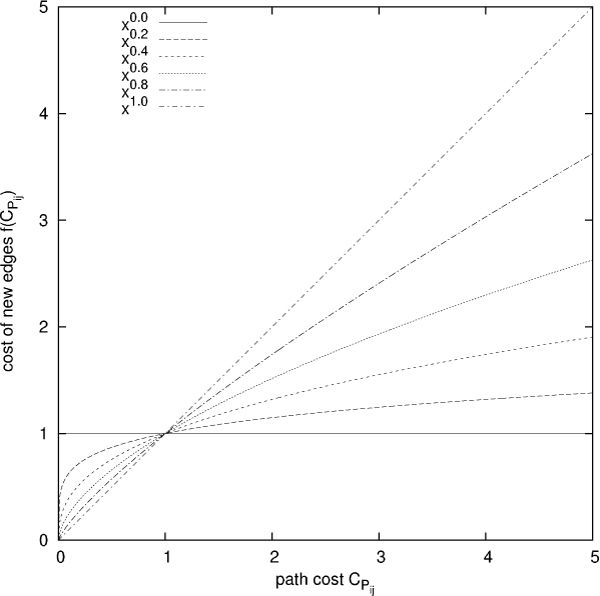
**Cost transformation function**. Different variants of the cost transformation function *f*(*C*_*Pij*_) for different values of *h *– "pathway persistence" (marked by different functions x^1-h^, h = 0.0, 0.2,...1.0). Under h = 1 the effect is maximal, since the cost of adding a new path that belongs to a known pathway does not depend on the length of the path and is always equal to 1.0. Under these conditions the search will stay within known pathways only.

In order to evaluate the approach we applied the promoter analysis algorithm (see Methods) to the promoters of genes encoding various known signal transduction pathways. Promoter sequences were extracted from the TRANSPRO^® ^database [16]. We were also testing a hypothesis that genes encoding components of the same signaling pathway are co-regulated by the "target transcription factors", i.e. TFs that receive the signal through the pathway in focus and, through that, provide multiple positive feedback loops in the pathway. We made this analysis for pathways and chains collected in the TRANSPATH^® ^database. Chains represent experimentally proven sequences of reactions and are utilized in the pathway search algorithm using nonlinear cost function (Figure 1).

We applied the *Match*™ tool [17] with default parameters (vertebrate matrices, minSUM cut-offs) and searched for potential TFBS in promoters of the genes encoding components of signal transduction pathways (see Methods). After that, we applied F-Match to compare site frequency in promoters of each pathway compared to the promoters of all other pathways. We found overrepresented sites for target TFs in 13 pathways and 23 chains (see Table 1). In the cases where the chains constitute a part of the same pathway, the sites were grouped together. Only 7 pathways and 2 chains exhibited underrepresented sites for their target TFs.

**Figure 2:**
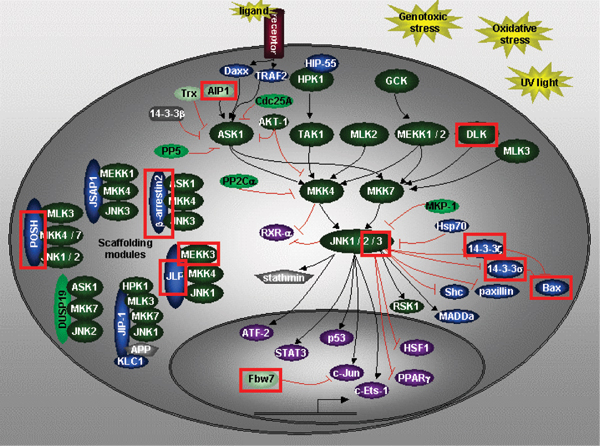
**JNK pathway**. With the red rectangle we marked molecules in whose gene promoters we can identify AP-1 sites – sites for the "target" TFs of the pathway. As one can see, such TF sites can provide fine regulation of the pathway activity through numerous feedback loops.

**Figure 3:**
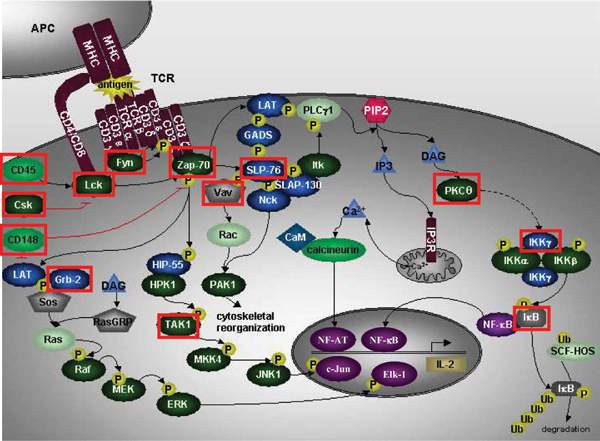
**T-cell antigen receptor pathway**. Sites for Elk-1 factor are overrepresented in the promoters of the genes from this pathway. Most of the hits are located at the upper part of the pathway suggesting the numerous feedback loops in the pathway.

We exemplarily show the results for two pathways (see Figure 2 and Figure 3). Sites for AP-1 factors have been found being over-represented in promoters of genes encoding components of **JNK pathway**. AP-1 sites were found in promoters of several genes encoding different variants of JNK kinases as well as other molecules upstream and downstream in the pathway leading to the activation and repression of the AP-1 transcription factors (see Figure 2). Such complex organization of the regulatory circuits clearly provides mechanisms for the autoregulation of the pathway. Evidence for direct autoregulation was found also for the pathways and networks activating the following TFs: p53, HIF1-alpha, p300, SRF, c-jun, NF-AT and DP1.

In the case of **T-cell antigen receptor pathway **sites for transcription factors Ets/Elk-1 are overrepresented in the promoters of the genes of this pathway. Most of the hits are located in the "upper" part of the pathway, e.g. molecules located in the pathway several steps upstream from the target transcription factors at the "bottom" (see Figure 3). We speculate that such organization of feedback loops from regulated TFs to the topmost parts of the pathway can provide optimal autoregulation, and enhances the specificity of the transduced signal.

**Figure 4:**
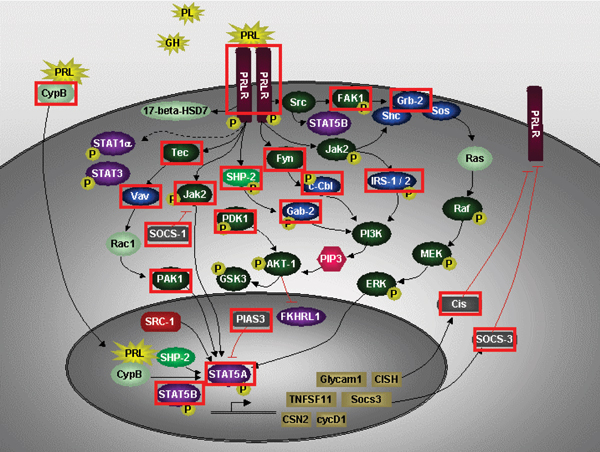
**Prolactin pathway**. Numerous sites for the "target" transcription factor FKHRL1 can be found in promoters of many genes encoding components of this pathway.

In the extreme case of the **prolactin pathway**, more than half of the genes in the pathway are probably regulated by the target TF FKHRL, including both the membrane receptor molecule and the target TFs STAT5A and STAT5B (see Figure 4).

**Figure 5:**
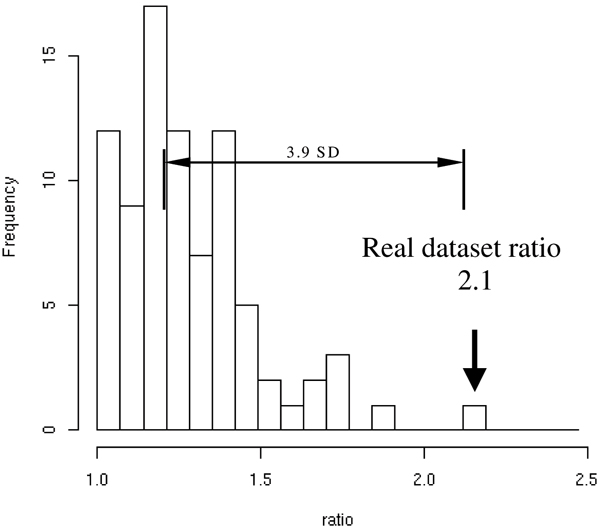
**Evaluation of significance of Elk-1 sites overrepresentation**. Histogram of the ratio between Elk-1 site frequency in two sets of promoters randomly chosen from TRANSPRO^® ^database. (100 times: 65 promoters in one set and 990 promoters in another set). The site frequency ratio 2.1 observed in the real set of promoters from T-cell antigen receptor pathway is 3.9 SD away from the mean of the distribution.

Since the PWMs used for identification of TFBS are relatively short, the chance that they can match with any DNA sequence is very high. The essence of our method is therefore to estimate the statistical significance of over- or under-representation of the number of matches in a given set of promoters (see Method section) as an indicator of potential functional involvement of TFs in the regulation of the corresponding genes. Here, we did an evaluation of the statistical significance of overrepresentation of Elk-1 sites found in the promoters of **T-cell antigen receptor pathway**. Two datasets were built collecting human promoter sequences at random from the TRANSPRO^® ^database, one with the same number as in the T-cell antigen receptor pathway (65 promoters), and another with as many promoters as in the control set (990 promoters). Elk-1 sites were predicted in both sets using the Match™ program, and their frequencies compared using F-Match. This simulation experiment was repeated 100 times, and a distribution of the ratio of Elk-1 site frequency in the query vs. control set was generated (see Figure 5). The average ratio of this random promoters distribution is 1.28 with a standard deviation of 0.21. Whereas, the ratio observed in the real set of promoters of **T-cell antigen receptor pathway **is 2.1 (we found Elk-1 sites in 13 promoters, but random expectation is 6.2 promoters), which is 3.9 SD units away from the random expectation (see Figure 5).

### Identification of key node TFs in the TRANSPATH^® ^pathways

**Figure 6:**
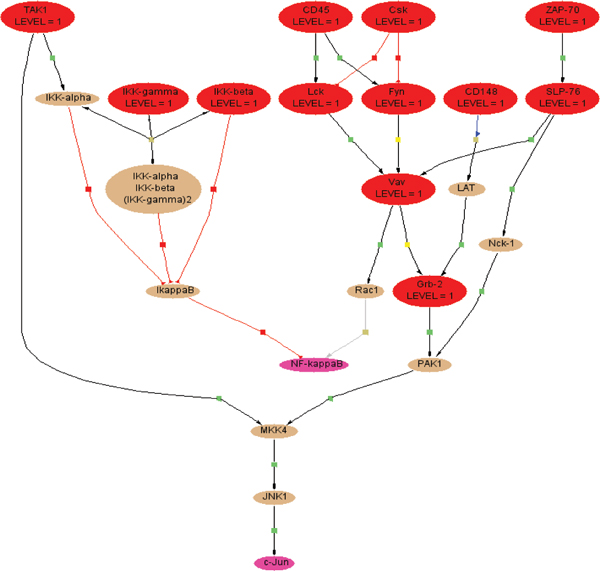
**Search for key TFs in T-cell antigen receptor pathway**. Automatically constructed diagram of the result of the search for key transcription factors. Starting from 12 molecules (out of 13) belonging to the T-cell antigen receptor pathway the search arrives at two TFs: NF-kappaB and c-Jun (AP-1) – known target TFs of this pathway.

To evaluate whether the "pathway persistence" parameter *h *can help in identifying key TFs, we applied the Pathway Persistence algorithm described in the Methods section to the **T-cell antigen receptor pathway **as it is presented in the TRANSPATH^® ^database (see Figure 3). As it can be seen from the diagram, 6 target transcription factors of this pathway are AP-1 (c-Jun), NF-AT, NF-kappaB (heterodimer of p50 and RelA), IkappaB and Elk-1. Out of 51 molecules belonging to this pathway we took 13 molecules whose gene promoters contain the Elk-1 binding sites (see Figure 3, molecules marked by red rectangles) identified at the previous step of analysis. We applied the key node search algorithm starting from these 13 molecules and searched downstream with parameters of the search: *dmax *= 4, *α *= (1/2)^12 ^; *h *= 0.9. The 7 top scoring transcription factors were: (E2F, IkappaB, NF-kappaB, PPAR-gamma, c-Jun, Egr-1, RXR-alpha). As one can see, NF-kappaB and components of AP-1 were among the top hits. Figure 6 shows the automatically generated output for the search of key TFs of the pathway.

**Figure 7:**
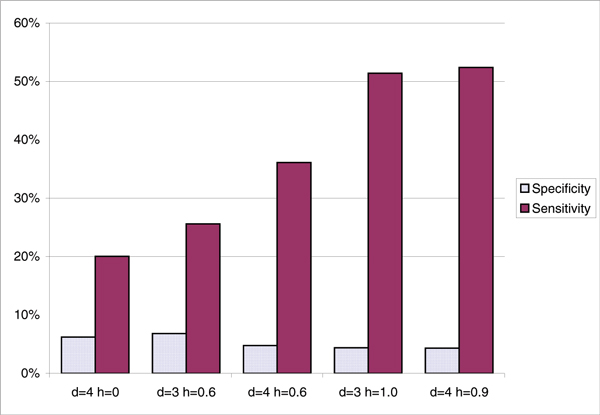
**The results of the simulation experiments**. Dependence of the sensitivity and specificity levels on the key node search parameters: *d*-distance, and *h*-pathway persistence. Usage of high values of persistence parameter *h *allows reaching sensitivity levels above 50% without significant decrease of specificity.

In order to evaluate the Pathway Persistence algorithm we made a series of random simulation experiments of the pathways analysis varying the parameters *dmax *(3, 4) and *h *(0.0, 0.6, 0.9, 1.0). For each pair of *dmax *and *h*, we randomly selected 10-times 13 molecules belonging to the **T-cell antigen receptor pathway **and performed the downstream search for the reachable TFs. We computed an average sensitivity (*Se*) and specificity (*Sp*) of the search by counting the average number of true target TFs (*N*) that were reached in such downstream search (out of the maximal number *N*_max _= 6), and by counting the average number of other TFs (*M*) that were also reached in the same search. Then:



The results of the simulation experiments are shown on Figure 7. One can see that the use of the high values of persistence parameter *h *allows reaching sensitivity levels above 50% without significant decrease of specificity.

### Analysis of TNF-alpha gene expression data

We applied the F-Match method (see Methods) to analyze promoters of differentially expressed genes in primary human endothelial cells upon TNF-alpha stimulation [15]. We compared promoters of genes induced or repressed by at least a factor of 2 (fold change, FC > 2.0) with the promoters of genes whose expression did not change upon this treatment. In this analysis, we were able to identify several PWMs whose hits are significantly over-represented or under-represented in the promoters in focus (see Table 2). We also compared results of identification of over- and under-represented sites taking different cut-offs of the expression fold change (FC>2.5 and FC > 3.0, see Table 2). We observed that most of the matrices identified at the FC cut-off 2.0, were also identified under the higher cut-offs and often the over-representation ratio was increasing, which clearly indicates the correlation between the gene expression change and the frequency of the corresponding sites in promoters. Some matrices have disappeared from the list of the significantly over- or under-represented sites (e.g., V$IRF_Q6 and V$MAZ_Q6) and several new matrices have appeared under the higher FC cut-offs (V$TBX5_02, V$HNF1_Q6, V$HFH3_01, V$HNF3B_01, V$CEBPGAMMA_Q6). Such matrices seem to be specific for a subgroup of promoters of the differentially expressed genes. We focus our further attention on the matrices that were identified under several FC cut-offs with increased stringency.

At the second step of the analysis we mapped all differentially expressed genes (with expression change higher than 2 times) onto the TRANSPATH^® ^database and extracted 129 signaling molecules (44 up-regulated and 85 down-regulated). Mapped molecules include all components of signaling pathways, such as receptors, their ligands, adaptor proteins, kinases of various levels as well as TFs. We applied the pathway analysis algorithm described in the Method section (downstream search with parameters: *dmax *= 4, *α *= (1/2)^12^; *h *= 0.9) and obtained a list of key transcription factors (see Table 3) characterized with the maximal score *s*_*i*_.

By comparison of the data in the Table 2 and Table 3 we can reveal TFs most probably involved in regulation of differential gene expression upon TNF-alpha stimulation. Among them are, e.g., NF-kappaB (and its inhibitor, IkappaB) and the components of AP-1 (c-Jun and c-Fos), the known targets of TNF signaling. It is interesting to observe that expression of genes encoding these two TFs has been changed in this experiment. For other factors such as FOXO, CREB, Elk-1 (ETS-domain), and E2F any significant change of their expression was not observed, but following the two independent sources of evidences, promoter analysis and pathway analysis, we conclude that they may play an important role in the regulation of gene expression in the considered system.

## Discussion

The approach demonstrated here allows identifying key TFs involved in the regulation of signaling pathways in the cell. The approach combines two algorithms, one is based on the analysis of promoter sequences and another on the analysis of the signal transduction pathway connectivity graph. As we demonstrated here, these two independent algorithms can often produce rather complementary or even similar results, pointing to the most important TFs involved in the regulation of the considered molecular processes.

The complementarity of the results of these two algorithms may suggest interesting dynamics of the considered pathways such as for the T-cell antigen receptor pathway analyzed above. First, we found an overrepresentation of sites for the ETS domain factor Elk-1 in the promoters of genes belonging to the pathway. Next, starting from these genes and searching downstream in the signal transduction network we identified other target TFs: NF-kappaB, AP-1 and NF-AT. So, we can speculate, that first signals coming through this pathway activate the Elk-1 factors, which in turn activates expression of many genes encoding components of the same pathway therefore enhancing the signal flow through this pathway. The amplified signals then activate other target transcription factors switching on the appropriate response in the cell.

A systematic analysis of many pathways reveals that sites for a total of 16 different TFs are overrepresented in the 36 pathways and chains, implying that different pathways share the same target TFs. We think that the fine-tune regulation of genes in overlapping or interconnected pathways might be achieved through the cooperative simultaneous regulation of many TFs, suggesting that a composite module analysis will further explain the differential regulation of these intersecting pathways.

## Conclusion

We developed a novel computational approach for revealing key transcription factors by knowledge-based analysis of gene expression data with the help of databases on gene transcription regulation and signal transduction networks, TRANSFAC^® ^and TRANSPATH^®^. We demonstrated that promoters of genes encoding components of many known signal transduction pathways are enriched by binding sites for transcription factors that are endpoints of the considered pathways. The corresponding software and databases are available at .

## Methods

### Microarray data

We have analyzed microarray gene expression data on TNF-alpha stimulation of primary human endothelial cells (HUVEC) [15]. Data have been taken from GEO (GSE2639). Gene expression profiles were measured in HUVEC stimulated for 5 hours with TNF and in untreated HUVEC using the Affymetrix^® ^GeneChip^® ^Human Genome U133A array [15]. Four biological replicates were used for each condition. We applied the criteria of an at least 2.0-fold change in gene expression levels and p-value revealed by t-test of less than 0.05. Based on these criteria, the expression of 121 transcripts was increased after TNF-alpha treatment and 214 transcripts showed decreased expression.

### Promoter sequences

Promoter sequences were taken from TRANSPRO^® ^[16], a database based on the international Genomic Sequence Builds for *H. sapiens, M. musculus *and *R. norvegicus*. We have collected information about TSSs from several databases, EPD [18], dbTSS [19] and Ensembl [20]. Collected TSSs are frequently not located in tight clusters, but widespread throughout the sequence. An algorithm applying a set of rules was designed to find 'clusters' of TSSs. A 'clustering score' was calculated by adding up contributions from each TSS in a sliding window, weighting the different reliability of TSSs from different sources and the distance of data points from the central window position. The peaks of the resulting clustering score function were regarded as potential 'virtual TSSs' for windows with enough evidence points. In cases of genes with only few data points the 5' most data point was used as 'virtual TSS'. This collection of 'virtual TSSs' is the basis for the extraction of promoter sequences.

### Association between promoters and signal transduction pathways

TRANSPATH^® ^[14] is a manually curated database focusing on signal transduction pathways. Relevant elements such as ligands, receptors, enzymes as well as TFs are stored along with information about interactions, modifications, and other reactions they undergo. Chains are sequences of reactions described in at least one paper, and several chains may constitute a pathway. There are 24441 promoters for 14283 human genes documented in TRANSPRO^® ^3.1, of them 1049 genes are associated with a pathway or reaction chain described in TRANSPATH^® ^7.1 [14]. These 1049 promoter regions -1000 to +100 relative to TSSs calculated in TRANSPATH^® ^were taken as a primary data source. The promoters are associated with 360 pathways or chains, provided that at least 5 promoters were associated with each pathway.

### Identification of putative TFBS

TRANSFAC^® ^10.1 provides, among other information, more than 8000 TFs, of them 1555 human TFs, as well as a library of 795 PWMs including 569 matrices for vertebrate TFs [1].

The program Match™ [17] was used to identify putative binding sites in the promoters of each pathway or chain, using the vertebrate PWMs. A built-in matrix profile with cut-off values adjusted to minimize the sum of false positive and false negative errors (*minSUM*) was used in the detection of the sites.

### F-Match: Identification of over- and under-represented sites in promoter sequences

In order to identify TFBS overrepresented in the promoter sets, a control set was derived for each pathway or reaction chain; the complete data source was taken excluding only the promoters of the genes in the pathway being investigated. The Match™ program was used to identify TF binding sites in this control collection and the number of sites was taken as a background to compare with the number of sites found in the promoters from the pathway in question.

We have developed a new program, F-Match, which compares the number of sites found in a query sequence set against the control set. We assume, if a certain TF (or factor family), alone or as a part of a *cis*-regulatory module, plays a significant role in the regulation of the considered set of promoters, then the frequency of the corresponding sites found in these sequences should be significantly higher than expected by random chance. Often, the stringency of the interaction of this TF with their target sequences in the considered promoters is not known leading to the uncertainty in setting thresholds on the site searches using the Match™ program.

F-Match carefully evaluates the set of promoters and for each matrix tries to find two thresholds: one, *th-max*, which provides maximum ratio between the frequency of matches in the promoters in focus (set A) and background promoters (set B) (over-represented sites); and the second threshold, *th-min*, that minimizes the same ratio (underrepresented sites).

In order to find these two thresholds, first, we apply the Match™ program to both sets of promoters using the lowest PWM thresholds corresponding to the minimum of false negative rates (minFN, see [17] for details). As a result, for each PWM we obtain a set of predicted *K *sites and *M *sites in the promoter sets A and B with the corresponding matrix scores *ms*:

*ms*_*A,1*_, *ms*_*A,2*_, ... *ms*_*A,K*_;

*ms*_*B,1*_, *ms*_*B,2*_, ... *ms*_*B,M*._

The F-Match program makes an exhaustive search through the space of all scores observed in the sequence sets. Each observed score is taken as a threshold *th *and the program computes the number of sites *k *found in the promoter set A (*ms*_*A,j *_≥ *th*/*j *= *1,K*) and number of sites *m *found in the promoter set B (*ms*_*B,j *_≥ *th*/*j *= *1,M*).

Then, the expected number of sites in the set A to be observed in the case of even distribution of sites between two sets will be:



and assuming a binomial distribution of the sites between two sets we can calculate the p-value of finding the observed number of sites and higher, for over-represented matches, or lower, in the case of under-represented matches



giving the p-value of over- and under-representation of matches in the promoter set A.

For a given significance level *ξ *(e.g. *ξ *= 0.001) the F-Match finds such thresholds *th-max *and *th-min *that maximizes and minimizes, respectively, the ratio *k/k*_*exp *_provided that the p-value <*ξ*. If the required significance level cannot be reached for a given matrix, then the program reports that no optimal threshold can be found.

### Identification of key nodes in signal transduction networks

To understand the mechanisms of gene expression, microarray data should be analysed in the context of complex regulatory networks of a cell. Through such networks, different signals can converge to few key TFs involved in regulation of the large gene sets. We have developed a tool, integrated in the ExPlain™ computer system, that allows fast search for key transcription factors regulated by a signal transduction network. A network is defined as weighted graph *G *= (*V, E, C*), where *V *= *genes *∪ *molecules *is the set of vertices, *E *= *reactions *are the edges and *C : E *→ ***R***^+ ^∪{*0*} is the cost function that defines a non-negative value for every edge. In the simplest variant of the algorithm, the initial values of the cost functions for each direct reaction are taken = 1.0. So, the cost of any path through the consequent reactions will be equal to the number of reactions.

Dijkstra's shortest-path algorithm is the core of this approach. The algorithm of downstream search starts from each molecule of the set of molecules in focus (subset *V*_*x *_of *V*) (e.g. signalling molecules whose expression has changed in the microarray experiment) and construct the shortest-path to all nodes *i *of *V *being within a given radius of *dmax*. After evaluating all nodes of *V*_*x *_the algorithm calculates the number of visits *N*_*i *_for each node *i *of *V*. This corresponds to the number of molecules in focus that can transfer the signal to the node *i *within *dmax *number of steps. The values *N*_*i *_could already be used as a ranking score for potential key nodes, since *N*_*i *_corresponds to the number of differentially expressed molecules from the initial list *V*_*x *_that can transfer the signal to the node *i*. In the case that node *i *represents a transcription factor and is characterized by a high *N*_*i*_, then this transcription factor seems the most probable target and key regulator of the signal transduction system under study.

In order to calculate the "specificity score" we use the formula:

*s*_*i *_= *N*_*i*_/(1 + *αM*_*i*_)     (3)

where *M*_*i *_is the number of all other molecules in the whole network, which can reach node *i *within *dmax *and which are considered as "non-relevant nodes". By setting different penalty levels (*α *= [0,1]) user has an opportunity to adjust the balance between true positives and false positives while searching for the key nodes. In the Web implementation of the algorithm we offer 21 penalty levels that correspond to:



The values *M*_*i *_are pre-calculated for every node and radius.

### Pathway Persistence algorithm

In addition to the information about individual reactions TRANSPATH^® ^contains information about pathways and chains of consecutive reactions known to take place in certain cellular conditions. Any pathway *P *is defined by a graph *G*_*P *_= (*V*_*P*_, *E*_*P*_, *C*) which is a sub-graph of the graph G.

We use the information about chains and pathways to improve the accuracy of key node prediction, especially to diminish the false positive error. While searching, the priority is given to the potential paths that utilize annotated chains of reactions. Still, predictions beyond the known paths are allowed but with low priority.

Application of pathway information into a certain graph can be transparently modeled by introduction of additional transitive edges with specific costs, which results in a new graph *G'*. The pathway information is fully represented by these additional edges. The main Dijkstra algorithm remains unchanged in this model. The additional edges for one pathway *P *are generated as follows: Let *S*_*Pij *_be the graph of the shortest paths between *i,j *∈ *V*_*P *_within the graph (e.g. pathway) *P*. Further, let *C*_*Pij *_be the cost of the shortest path with the exceptional case where *C*_*Pij *_= ∞ if *S*_*Pij *_= ∅. We combine *G*_*P *_and *G *yielding *G' *= (*V*,*E'*,*C'*) simply by introducing new edges *E' *= *E*∪{(*i,j*) | *S*_*Pij *_≠ ∅} and by extending the cost function for them by *C' *= {*f*(*C*_*Pij*_) | *f*(*C*_*Pij*_)≤*C*_*ij*_}∪{*C*_*ij*_|*f*(*C*_*Pij*_)>*C*_*ij*_}. As function *f *we use *f*(*x*) = *x*^(*1-h*)^, with *h*∈ [*0,1*]. The aim of *f *is to make the cost function of a new edge (*i, j*) sublinearly dependent on the length of the corresponding shortest path *S*_*Pij *_within each pathway. As a result, with increasing *h *the shortest-path search is progressively pushed to stay within known pathways (see Figure 1). This affects the search of the key nodes. The effect is maximal if *h *= *1 *and absent if *h *= *0*. Therefore we call *h *"pathway persistence" which allows the user to select the proper balance between making speculative predictions or staying within known pathways only. This approach is inspired by Higher-Order-Markov-Chains, where subsequent past states define the probability of the future states of a stochastic system.

Duplicated edges can occur due to pathway overlapping. We merge them to one edge by combining their costs synergistically. The cost of the merged edge is calculated from the individual costs *c*_*i *_by



While the search for common key nodes is already easily possible by application of Dijkstra to *G'*, the connection details between a certain identified key node and starting nodes are not yet obvious, since they are established in *G'*, not in *G*. Therefore, we implemented a sophisticated dynamic cost function which still can be used by Dijkstra, and which mimics the existence of edges *E' *on-the-fly, while the shortest-paths themselves consist of edges from *E *only.

## List of abbreviations

TFs – transcription factors

HMMs – Hidden Markov Models

PWMs – positional weight matrices

TFBS – transcription factor binding sites

TSSs – transcription start sites

## Authors' contributions

AKE carried out analysis of the pathways, participated in development of promoter analysis algorithm and drafted the manuscript. NVO developed the algorithms for pathway analysis. RJA carried out the analysis of promoters of genes from signal transduction pathways and prepared the figures and tables in the manuscript. OKE participated in the design of the study, discussion of the results and performing systematic data collection. EWI participated in coordination and design of the study and helped to draft the manuscript. All authors read and approved the final manuscript.

## Figures and Tables

**Table 1:** Over and under-represented sites in pathways. Pathways in which over- and under-represented sites were identified for "target" transcription factors – TFs that receive the signal through the pathway in focus. Corresponding PWMs are shown.

**Pathway in TRANSPATH^®^**	**TF (family)**	**TRANSFAC^® ^PWM acc**	**p-value**
***Overrepresented sites in pathways***			

mTOR --> S6& eIF-4E	STAT	M00777	9.51E-003
MHC class II -> NF-AT	NF-AT	M00935	1.92E-004
IFNgamma -> STAT1alpha	STAT	M00777	6.27E-003
BCR -> c-Jun	AP-1	M00517	8.91E-003
BCR -MLK3-> c-Jun	AP-1	M00517	6.61E-003
TNF-alpha -> NFkappaB	NF-kappaB	M00774	9.86E-003
VEGF-A -> NF-ATc	NF-AT	M00935	7.31E-003
15d-PGJ2 -> PPAR-gamma	p300	M00033	7.16E-003
G1 phase (Cdk4)	E2F	M00738	9.53E-004
S phase (Cdk2)	E2F	M00738	6.96E-006
IL-1beta -> c-Jun	AP-1	M00517	2.89E-003
MKK6 -> p53	p53	M00761	8.64E-003
MKP-5 -/p53; MKK4 -> p53	p53	M00761	7.47E-003
Caspase-3 -/p53	p53	M00761	9.81E-003
p53 pathway	AhR	M00976	7.59E-003
HIF-1alpha pathway	AhR	M00976	2.34E-003
IL-1 pathway	AP-1	M00517	6.57E-003
E2F network	E2F	M00738	7.73E-006
T-cell antigen receptor pathway	Ets/Elk-1	M00971	7.44E-005
TLR4 pathway	p300	M00033	4.29E-003
stress-associated pathways	Ebox; AP-1	M01034; M00517	5.96E-03; 1.26E-03
EDAR pathway	NF-kappaB	M00774	8.90E-004
wnt pathway	Ets	M00971	7.10E-003
TNF-alpha pathway	NF-kappaB	M00774	9.03E-003
EDA-A2 -TRAF3-> NF-kappaB	NF-kappaB	M00774	2.31E-004
EDA-A1 -> NF-kappaB	NF-kappaB	M00774	2.31E-004
dsRNA -> IRF-7:IRF-3:CBP:p300	p300; IRF	M00033; M00972	6.90E-03; 2.30E-03
TLR3 pathway	NF-kappaB	M00774	5.27E-004
dsRNA -> p50:RelA	NF-kappaB	M00774	1.35E-003
LPS -> IRF-3:IRF-7:CBP:p300	p300	M00033	4.86E-004
p38 pathway	STAT; Ebox; CREB/ATF	M00777; M01034; M00981	5.88E-04; 5.14E-03;2.33E-03
JNK pathway	AP-1	M00517	9.24E-003
p38alpha -> MITF	Ebox	M01034	9.11E-003
Fas -/SRF	SRF	M01007	6.57E-004
PRL pathway	STAT; FOX	M00777; M00809	7.43E-03;6.85E-03
PRL -PI3K-> AKT	FOX	M00809	1.97E-005

***Underrepresented sites in pathways***			
Rac1 -> c-Jun & Elk-1	AP-1;Ets	M00172; M00971	4.92E-03;3.55E-03
insulin -> AKT-1 pathway	CREB	M00917	6.83E-003
EGF pathway	AP-1	M00926	3.03E-003
insulin pathway	CREB	M00917	3.45E-003
Rac1 pathway	AP-1; Ets	M00172; M00971	8.14E-03; 7.46E-03
stress-associated pathways	Ets	M00971	8.44E-003
p38 pathway	STAT	M00777	1.70E-005
E1 -PIRH2-/p53	p53	M00761	6.57E-003
hypoxia pathways	p300	M00033	6.45E-003

**Table 2:** Over and under-represented sites in in TNF*α *expression data. Predicted sites that are significantly over-represented (Ratio [FC>2.0] > 1.4) and under-represented (Ratio [FC>2.0] < 0.7) in the promoters of genes that are differentially expressed in primary human endothelial cells upon TNFα stimulation

**Matrix ID in TRANSFAC^®^**	**Ratio [FC>2.0]**	**#sites *k***	**#sites *m***	**p-value**	**Ratio [FC>2.5]**	**Ratio [FC>3.0]**
V$NFKAPPAB65_01	**5.54**	36	7	1.45·10^-6^	**10.44**	**12.49**
V$CREL_01	**3.29**	58	19	1.04·10^-6^	**4.83**	**5.52**
V$CDX2_Q5	**2.45**	41	18	7.35·10^-4^	**2.90**	**2.74**
V$IRF_Q6	**1.88**	68	39	9.46·10^-4^	**-**	**-**
V$AP1_01	**2.09**	35	18	6.51·10^-3^	**2.55**	**2.78**
V$FOXJ2_02	**1.58**	94	64	2.70·10^-3^	**1.61**	**1.78**
V$MAZ_Q6	**1.45**	284	211	2.32·10^-5^	**-**	**-**
V$GATA4_Q3	**1.58**	139	95	3.50·10^-4^	**2.54**	**2.69**
V$CETS1P54_01	**0.20**	6	32	3.19·10^-5^	**0.38**	**0.50**
V$YY1_02	**0.30**	12	43	5.21·10^-5^	**0.25**	**-**
V$USF_02	**0.38**	18	50	2.15·10^-4^	**0.36**	**0.39**
V$CREBATF_Q6	**0.61**	65	114	9.35·10^-4^	**-**	**-**
V$E2F_Q3_01	**0.63**	88	149	4.13·10^-4^	**0.36**	**0.28**
V$MYC_Q2	**0.64**	87	145	7.05·10^-4^	**0.45**	**0.50**

*k *and *m *– numbers of sites computed in A and B sets respectively. p-value is computed according to the eq. 2.

**Table 3:** Key transcription factors found by pathway analysis of TNF*α *expression data. Transcription factors found by the downstream key node search starting from 129 signal pathway molecules whose expression has been changed more than 2-fold in primary human endothelial cells upon TNFα stimulation. (requires some explanation of the parameters shown here) Ni - number od relevant reachable molecules from the key node; Mi - number of non-relevant reachable molecules from the key node; Si - specifically score (see equation (3)).

**TRANSPATH^® ^molecule acc**	**TF name**	***N***_***i***_	***M***_***i***_	***s***_***i***_
MO000018096	MEF-2C	80	1520	67.48
MO000020766	MEF-2°	71	557	66.48
MO000020759	NF-AT2	82	2040	65.65
MO000016887	STAT1alpha	89	2920	65.61
**MO000000279**	**c-Fos**	**83**	**2202**	**65.42**
**MO000017496**	**FOXO3**	**74**	**1292**	**63.92**
MO000013133	STAT5B	84	2578	63.89
MO000033504	PPAR-alpha	73	1218	63.55
MO000013122	STAT3	94	3946	63.44
MO000037096	MITF	78	1955	62.97
MO000019430	ER	72	1357	61.77
**MO000017189**	**CREB**	**86**	**3223**	**61.72**
**MO000000038**	**Elk-1**	**80**	**2427**	**61.72**
**MO000038332**	**IkappaB-alpha, IkappaB-beta**	**87**	**3562**	**60.64**
**MO000045008**	**FOXO3, FOXO4**	**78**	**2350**	**60.61**
MO000017212	NF-AT	82	2906	60.53
**MO000044995**	**FOXO**	**78**	**2370**	**60.5**
MO000013119	STAT1	87	3600	60.44
MO000022492	PPAR-gamma	77	2269	60.3
**MO000017515**	**E2F**	**72**	**1638**	**60**
MO000013126	STAT5A	82	3035	59.83
MO000000062	ATF-2	73	1818	59.74
MO000016628	SAP-1	77	2407	59.51
MO000000059	SRF	80	2885	59.16
MO000016876	STAT5	84	3521	58.75
MO000018995	RXR-alpha	77	2551	58.72
MO000034151	HIF-1alpha	78	2762	58.33
**MO000045358**	**YY1:p53**	**71**	**1879**	**57.75**
MO000017658	Smad2	82	3513	57.39
**MO000000049**	**c-Jun**	**82**	**3622**	**56.86**
**MO000000058**	**NF-kappaB**	**77**	**3132**	**55.7**
